# Exclusive breastfeeding and its association with intimate partner violence during pregnancy: analysis from Pakistan demographic and health survey

**DOI:** 10.1186/s12905-024-02996-2

**Published:** 2024-03-20

**Authors:** Neelum Khalid, Zhongliang Zhou, Rashed Nawaz

**Affiliations:** School of Public Policy and Administration, Xi’an Jiaotong University, Shaanxi, PR China

**Keywords:** Intimate partner violence, Exclusive breastfeeding, Infant feeding, Domestic violence, Pakistan

## Abstract

**Background:**

Intimate partner violence (IPV) is a global social issue and increasingly asks for the attention of policymakers. IPV is one of the main factors that affect the health of pregnant women and their infants during pregnancy and after childbirth; it will not only cause direct harm to women themselves but also reduce women’s exclusive breastfeeding (EBF) behavior and pose a threat to newborn health. Existing facts on the association between IPV and EBF in the Pakistani context are negligible and incomplete to an enduring measure of IPV practice. To this effect, the present study aims to investigate the relationship between EBF and IPV practiced during the prenatal period and post-delivery.

**Methods:**

The statistics study has drawn from the Pakistan Demographic and Health Survey (PDHS) 2018. A total of 1191 breastfeeding females aged 15–49 with children under 6 months were selected for the present study. T-test or chi-square test of Univariate test of hypothesis; Logistic regression model was utilized to explore the potential impact of IPV on female exclusive breastfeeding from three dimensions of physical, sexual and psychological violence, to provide data support for the Pakistani government to formulate policies to promote female EBF. All investigations have been performed in STATA software 16.0 (Stata Corp, College Station, TX, USA) at 95% confidence interval.

**Results:**

Among the 1191 participants, 43.6% (520 / 1191) of the females were EBF, while the rates of physical, sexual, and emotional IPV were 47.44%, 30.23%, and 51.72%, respectively. Logistic regression analysis showed that females who have experienced physical IPV were 32% less likely to be exclusively breastfed (aOR = 0.68; 95% CI; 0.490, 0.980; *P* < 0.05), the chances of EBF were reduced by 22% in women who experienced IPV (aOR = 0.78; 95 CI; 0.55, 1.00; *P* < 0.05), females who experienced emotional IPV were 31% less probable to exclusively breastfed (aOR = 0.69; 95% CI; 0.47, 0.92; *P* < 0.05).

**Conclusions:**

This study determines the adverse effects of sexual and psychological violence on EBF practices in women. Policymakers in Pakistan should actively implement assistance programs to reduce IPV, emphasize monitoring women’s experiences of IPV before and after giving birth, and encourage women to break the “culture of silence” when they experience IPV to maximize their access to assistance.

## Background

The advantages of breastfeeding have been proven to be effective. Connect to the constructive health results regarding the mother and infant. Epidemiological proof has demonstrated that breastfeeding decreases the danger of digestive and breathing tract impurities between babies [1]. Breastfeeding is the finest foundation of nourishment for newborns as it is pure and encompasses every vital nutrient essential for infants in their early birth stage. The beginning of breastfeeding after the first hour of delivery is about 50% in many emerging nations [2]. According to the WHO guidelines, the proportion of the EBF practice is considerably lesser, and just 38% in Pakistan [3]. EBF signifies nourishing a newborn only with breastmilk for six months of birth [4, 5]. The main advantage of EBF practice, particularly for newborns, has been to strengthen the immune system and minimize the hazard of morbidity [6–8]. Breastfeeding supports avoiding hypothermia and hypoglycemia among infants, and wherever the utmost newborn mortalities are an outcome of the contaminations, nourishing colostrum and predominantly exclusively breastfeeding the newborn has been established to be shielding in contradiction of such demises [9]. EBF plays a critical part in minimizing newborn deaths and delivering the nourishment to protect in contradiction of contaminations that safeguard existence. EBF is extensively acknowledged as vital to promoting infant healthiness and development [10]. Well-known advantages of breastfeeding contain an inferior occurrence of communicable and non-communicable sickness, diarrhea, respirational infections, and also ear contamination among the babies. EBF has also been identified to enhance newborn intellectual growth and improve and simplify new mothers’ child-rearing expertise [11, 12]. Nevertheless, whether to continue breastfeeding altogether pivots on numerous societal, emotional, psychological, and environmental features [13]. The mothers who have experienced IPV are used to adopt depressive signs or other serious well-being problems [14–16]. IPV signifies the exploitation or violence among individuals who are in a close relationship [17].

Studies have suggested that approximately one in three ever-married females in Pakistan are informed to have practiced physical, sexual, or emotional IPV, with a growing tendency noted in the previous three decades for all three forms of IPV [18–21]. IPV has also revealed that it has been related to the inferior standards of societal livelihood, more excellent standards of trauma, and hopelessness between the females of the reproductive stage [22, 23]. Additionally, IPV at the time of pregnancy is connected with deprived newborn well-being results, and it also bounds fresh moms’ capabilities and skills to cure their newborns constructed upon the husbands’ suspiciousness and refusal to reduce the physical and sexual desires of the females through the time of the post-delivery [24].

Investigation from the advanced nations has highlighted that the association among IPV and EBF has diverse outcomes. Prior researchers have also recommended that a newborn’s introduction to IPV may perhaps pose a hazard of distress or psychopathology during initial infancy. Advanced nation’s investigations have scrutinized the association among the IPV and EBF conveyed diversified outcomes. For example, the connection between IPV and limited EBF has been highlighted by researchers from Spain and the United States of America (USA). No such connotation among IPV and EBF has been highlighted in the research from Australia and Sweden [25–27]. The variations in the outcomes of different countries could be reasons for alterations in the category of the statistics utilized. Some outcomes of the research have been the utilization of the nationwide investigation. In contrast, several other studies have been centered upon the statistics concerning members in a database at a healthcare organization. But from the further prospective research from the emerging nations, precisely the studies through South Asia have frequently testified to the connotations [28–30]. From the perspective of sub-Saharan Africa, sole research has scrutinized the connotation of IPV and EBF. The said research was based on a comparative investigation involving 8 African nations. The outcomes of Nigeria have demonstrated no modified connotation among all types of IPV and EBF practices, which were dignified through lifetime practice [31].

With respect to the period of the occurrence, IPV might be of several variations, for instance, lifetime practice, pregnancy involvement, or post-delivery experience. Violence practiced in earlier eras might not have such adverse outcomes as that come across at a more proximal interval of the breastfeeding stage. Female education, an important indicator of women’s empowerment, highlighted the huge imbalance in Pakistan. According to a study, about half of women in Pakistan have shown a lack of primary education [32]. that information regarding the association among IPV and EBF in Pakistan is to be wholly recognized, the present research has focused on re-examining this association, having emphasis upon IPV measured from the gestational period and postpartum experiences. Correspondingly, such experience has been experimented with to what extent disturbing nursing responsibilities might influence the features of the research population. Consequently, due to the significance of the fact that information regarding the connection between IPV and EBF practices in Pakistan has not been completely recognized, the current research purpose has been to examine the association by emphasizing IPV during and after pregnancy. Current research outcomes postulate that generally, women experienced violence (physical, emotional, and sexual) for almost one time in their whole life and the current proportion is much greater inside less developed regions concerning the metropolitan regions. Earlier research has specified that an inferior standard of a couple’s schooling, joblessness, and deprived socio-economic situations have a straight association between early-age weddings and partner violence [33].

## Methods

### Data source and research design

The DHS Database is a program supported by the United States Agency for International Development (USAID). The present research utilizes data from PDHS 2018. Overall, 1391 females have been carefully chosen. All mothers aged between 15 and 49, and men aged between 15 and 59, who were either legal residents or guests at conference time, have been entitled to participate. Queries have been enquired that were linked to household socio-demography, motherly well-being, and along with infant health. The IPV module has been a subsample module inside the investigation and has been constructed upon a summarized and adapted form of the Conflict Tactics Scale (CTS) [34]. The original scale was modified between 1998 and 1999 by ICF Macro, DHS investigation controller, and afterward, discussion with the professionals concerning internal violence measures, gender, and study investigation [35]. This has been successfully verified and authenticated with the help of pilot investigations in Cambodia and Haiti in the years 2000 and 2002 in the Dominican Republic [35]. Synchronized rationality was also built for the modified scale. Then, it was utilized to implement DHS programs in more than 90 nations and diagonally in Africa, Asia, and Latin America. Furthermore, researchers using the statistics from these surveys have constantly informed a greater Cronbach’s alpha value that indicates an interior consistency of concept [36, 37]. The DHS agenda’s adapted CTS comprises details that include queries regarding sexual violence together with physical violence and should make no assumptions that violence occurs in conditions created during the conflicts. Single-appropriate females in every domiciliary have been casually nominated to enquire supplementary interrogations regarding household violence. The IPV sub-sample selection pattern that has been translated from English into Urdu and Sindhi has been made nationally demonstrative in particular by utilization of constructed weights. demonstrative. Three precise protections have been added to the survey inquiry form by keeping in mind ethics and security commendations as per guidelines of the World Health Organization (WHO) [38].

**Fig. 1:**
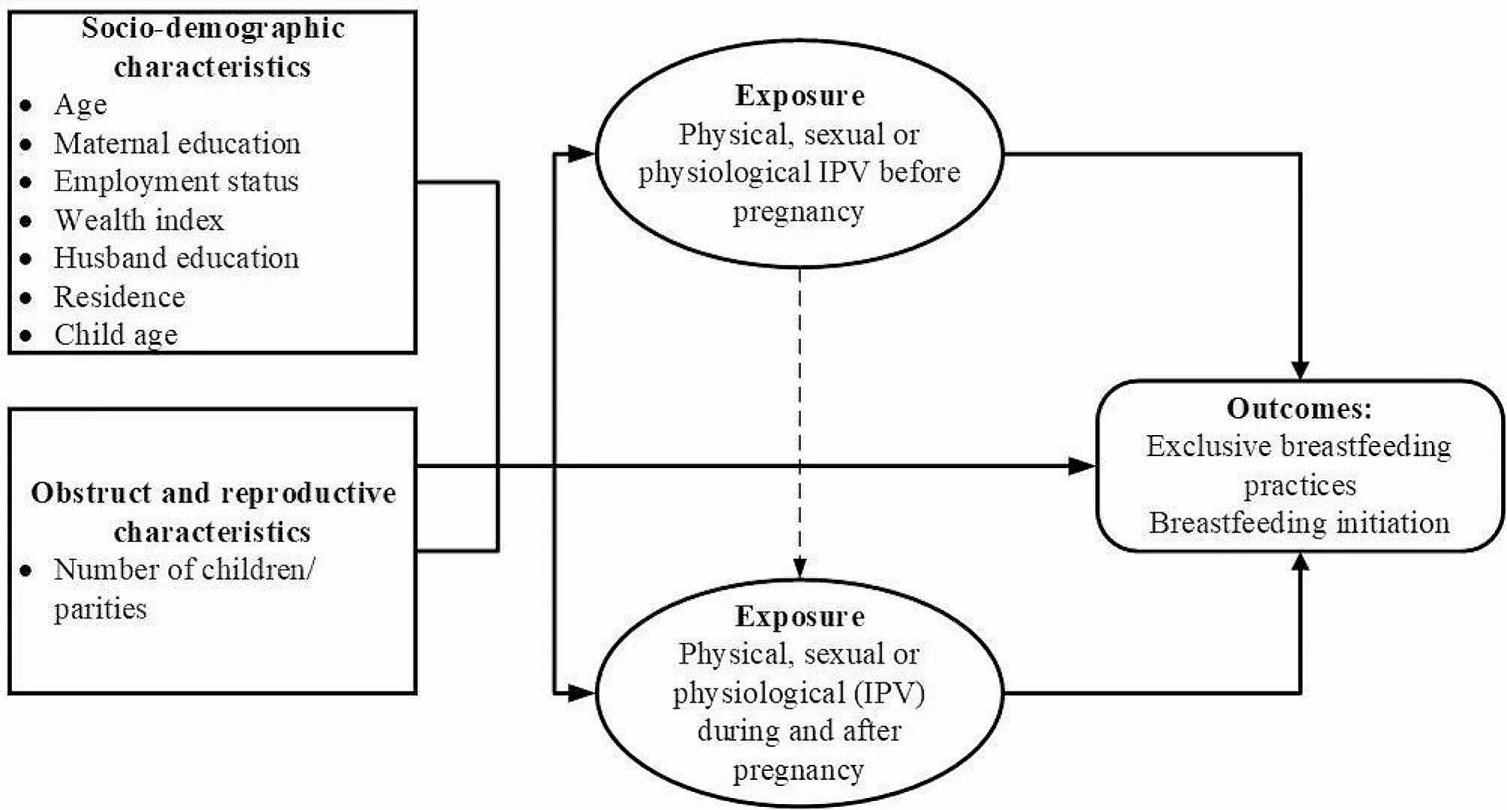
Theoretical framework for the influence of IPV before, during, and after the pregnancy and EBF practices

Figure 1 of the current study highlighted the EBF practices and their association with IPV during pregnancy; the conceptual framework presents the connotation among the independent and dependent variables. The current research utilized IPV as an independent variable; EBF practices and breastfeeding initiation have been used as the dependent variables. Meanwhile, age, education level, employment status, husband education, wealth, IPV, residence, parity, and marriage type were used as control variables.

### Research study indicators

The outcome variable of the present study is binary, demonstrating that a breastfeeding infant was less than 6 months going through EBF (= 1) or mixed-feeding (= 0). It has been kept in knowledge through questions on whether the infant has been provided the specific forms of food or semi-solid nutrients in advance twenty-four (24) hours of the survey. These types of solids and semi-solid items were not recommended for a child whose age is less than 6 months. Those respondents who have not nourished the infant with registered nourishment solids other than breast milk have been observed practicing EBF. The exposure variables have been the form of IPV investigated by utilizing 13 questions enclosed inside the DHS plan’s adapted CTS acknowledged as a domestic violence component of DHS [35]. It has been based upon probable violent actions a mother has practiced through her present man during the pregnancy and after delivery has been highlighted in Table 1. It has been supposed that interval-captured events might have happened during pregnancy or post-delivery. The questions have been gathered around 3 types of IPV. Psychological IPV consisted of 3 interrogations (questions 1–3) having Cronbach’s alpha value of 0.75. Physical IPV has contained 7 inquiries (questions 4–10) having Cronbach’s alpha value of 0.86. At the same time, sexual IPV has also contained 3 interrogations (interrogations 11–13) that have Cronbach’s alpha value of 0.85. A collective factor has been shaped which contained the practice of any IPV. Every item has been binary coded. Furthermore, the occurrence of the IPV has been created through the 13 probable events. It has been utilized to quantify the amount of an IPV event. The queries inside the examination have been enquired regarding the occurrence of violence. The answers comprise; Never= (0), Sometimes= (1), and so on Often= (2). Consequently, this has produced a scale from 0 (no violence has been done during the previous 12 months) to 26 (undergoing each violent occurrence every so often in the last 12 months).

**Table 1:** Interrogation of variables and responses design on Intimate Partner Violence (IPV)

**Serial number**	**Items**	**Responses**	
		**Yes**	**No**
1.	Does your spouse/companion ever disgrace you	1	0
2.	Have you been endangered with injury from your spouse	1	0
3.	Has your spouse/partner ever disrespected you	1	0
4.	Have your spouse/partner has strapped, traumatized, and frightened you by throwing something at you	1	0
5.	Have your spouse/partner has ever slapped you	1	0
6.	Have your spouse/partner pressed you or knocked you with something that harmed you	1	0
7.	Have your spouse/partner has jerked or pulled you	1	0
8.	Does your spouse/partner suppress or try to burn you	1	0
9.	Has your spouse/partner shown a blade/revolver or any other armament	1	0
10.	Has your spouse/partner Ever warped your arms and hair hauled your hairs	1	0
11.	Has your spouse/partner ever enforced you into undesirable sexual practice	1	0
12.	Does your spouse/partner has threatened you for undesirable erotic activity	1	0
13.	Does your spouse/partner enforced you substantially execute erotic activity, while you are not willing to do	1	0

A number of the covariates that have been adopted in current research were discussed in prior studies and have been probable factors of the EBF practice [31, 39, 40]. This consists of the infant’s age quantified in months; the mom’s age has also been quantified in years; the mother’s schooling has been listed as the total number of years of official schooling learned or has been acquired; parity has been quantified as the total babies have by a female; partner’s schooling has been developed as the total amount of years of proper schooling learned by the partner; the total quantity of other babies that were less than 5 years of age inside DHS was also quantified in sum total method; Household wealth index has been created by utilizing family capital information by the help of a prime module examination. The household wealth index item has been previously calculated and accessible as a portion of the DHS information. Additional covariates contained mothers’ employment status during the last year before the investigation was characterized, and the authors have considered the wealth index as two-fold (employed vs. not employed); metropolitan status has been assembled as binary (Urban vs. Rural) residence.

### Statistical analysis

We have utilized the sampling weights to describe the delivery of the independent factors, covariates, and EBF practices. Chi-square and t-test analysis have been utilized to accomplish the associations among the independent variables and dependent factors. For the association among the factors, we have used the chi-square test. Meanwhile, the mean values were calculated by utilizing a t-test. To overcome the issue of multicollinearity, the authors have directed an analytical check among the factors and control variables; the variance inflation factors (VIF) value was less than 10, having a 1.83 normal VIF value. Additionally, the authors have utilized two logistic regression examinations. The first was a comprehensive case examination with no missing observations (*N* = 1391). The following regression analysis contained several imputation investigations with absent observations (*N* = 1191). This was done to utilize the comprehensive evidence in the designated study sample fully. The current imputations have been performed by utilizing the procedure of Markov Chain Monte Carlo (MCMC) [41, 42] underneath the supposition of missing at random (MAR) [41]. The current study has quantified 11 imputations, which were considered satisfactory to reproduce the variance-covariance approximation according to the specifications of the Monte Carlo error check [41]. Every regression approach consists of five models; each model consists of one independent item with control factors. To conduct all investigations and data imputation as well, we have utilized Stata version 16. All regression examinations have been conducted by utilizing the study scheme (sampling weights). A 95% confidence interval has also described the Adjusted Odds Ratios (AOR).

## Results

### Basic characteristics of the sample

Table 2 explains that the average age of the women in the current research is 27.7 years, whereas the education level of the respondents is 4.5. The partners of the study had an average of 7 years of education. The overall age of the children in the current study is 3 years. The average age of the children in the current study was 3 months, the oldest was 6 months, and the youngest was 1 month old. Furthermore, Table 2 also represents the basic characteristics of the categorical variables and ratio distribution of the current research. The total number of females is 1191. The employment status of the whole sample has been divided into two categories. i.e. employed and the unemployed. Whereas the wealth index of the current study was divided into five categories: poorer, poorest, middle, richer, and richest., Furthermore, the parity status of the respondents has also two divisions, the primiparous and the multiparty. Additionally, the residence status has been also categories into urban and rural residence.) of the females were residing in urban areas where as the greater proportion of respondents (53.8) were from rural regions.

**Table 2:** Cross-tabulation of non-weighted basic characteristics of defendants by EBF practices

	**Complete**				**Exclusive Breastfeeding (EBF)**			
	**Nos**	**%**	**Nos**	**%**	**Yes**	**%**	**Mean**	**SD**
**Covariates**								
Women Age							27.7	7.5
Female’s Education							4.5	5.2
Husband Education							7	5.1
**Employment status**								
Currently Employed	528	44.7	312	47.7	186	34.7		
Not Employed	663	55.3	342	52.3	351	65.3		
**Wealth Index**								
Poorest	247	20.74	63	13.69	84	7.05		
Poorer	275	23.09	160	13.43	115	9.66		
Middle	244	20.49	170	14.27	74	6.21		
Richer	199	16.71	120	10.08	79	6.63		
Richest	226	18.98	146	12.26	80	6.72		
**Parity**								
Primiparous	180	15.2	138	23.1	156	26.7		
Multiparity	1011	84.8	460	76.9	435	73.3		
Child’s age							3	1.8
Nos. of under-five child’s							2.6	1.4
**Residence**								
Rural	643	53.9	389	52.7	254	55.9		
Urban	548	46.2	348	47.2	200	44.0		

Total Sample Size = 1391, Under-five child’s = child’s less than five years, SD = Standard deviation

### Univariate and bivariate analysis

The frequency of IPV in the research section has been 43.24%, psychological IPV has been testified to the utmost (51.72%), and sexual IPV has been reported as (30.23%). From another perspective, physical IPV had an occurrence of 47.44%. The normal incidence score of IPV amongst the defendants is 0.58 (SD = 2.0). Amongst the experience items, the chi-square bivariate has been solely directed toward a momentous affiliation among physical IPV and EBF at a 95% confidence interval in Table 3. Furthermore, the t-test information has been directed a noteworthy dissimilarity in the means of frequency score of IPV through clusters of EBF, EBF vs. mixed-feeding) 95% confidence interval, t-test = 2.03: *P value* < 0.05 highlighted in the Table 3. The age of babies has been adversely connected to EBF practice.

**Table 3:** Cross-tabulation of descriptive characteristics by EBF practice of respondents

**Variables**	**Complete**		**Exclusive Breastfeeding (EBF)**				**t/χ2**	**P**
	**Frequency**	**%**	**No**	**%**	**Yes**	**%**		
Emotional IPV							2.62	0.100
No	575	48.28	353	46.51	222	51.39		
Yes	616	51.72	406	53.49	210	48.61		
Physical IPV							3.70	0.050
No	626	52.56	383	50.46	243	56.25		
Yes	565	47.44	376	49.54	189	43.75		
Sexual IPV							1.58	0.200
No	831	69.77	520	68.51	311	71.99		
Yes	360	30.23	239	31.49	121	28.01		
Any IPV							0.00	0.980
No	676	56.76	431	56.79	245	56.71		
Yes	515	43.24	328	43.21	187	43.29		
Frequency score of IPV								
$\stackrel{-}{x}\pm sd$	1191	0.58 ± 2.00				†2.45	0.020	

Number of observations = 1191, Referred sampling weights, † t-test information, SD = standard deviation

### Demographical analysis

A demographical examination was conducted to discover the accuracy of the current research sample size. The evidence provided by the participants is highlighted in the form of a table so that it can be easily understandable. Table 2 explains the demographic characteristics of the defendants of the survey conducted. The table depicted the mean age of defendants as 27.7 years (SD = 7.5), the normal schooling in years that have been accomplished was 4.5 years (SD = 5.2), and a significant portion (55.3%) has never been engaged with work. Concerning responders’ productiveness, a superior amount (84.8%) has been multiparous, and the infants’ average age was approximately 3 months (SD = 1.4). A more significant percentage of the defendants (53.9%) were inhabitants of rural regions.

Furthermore, Table 4 explains detailed information about the age of the infants in months, exact percentage of the EBF, no form of IPV, and any form of IPV. The infants who were one, two, three, four, five, and six months have 84.10%, 82.20%, 70%, 54.50%, 43.20%, and 28.90% have EBF rates, respectively. Similarly, for infants aged 1–2 months, 48.60% of the womenfolk have practiced no IPV form, whereas for children aged 3–4 months, 49.10% of the females have practiced no IPV. Moreover, 60.30% of womenfolk have not practiced any form of IPV whose infant’s age was 5–6 months. Additionally, 51.10%, 50.90%, and 39.70% of the women have practiced any form of IPV, whose infants aged were from one to two months, three to four months, and five to six months, respectively.

**Table 4:** Rate of Exclusive Breastfeeding and Age of Infants

**Age of Infants**	**Exclusive Breastfeeding**	**No form of IPV**	**Any form of IPV**
1 Month	84.10%		
2 Months	82.20%	48.60%	51.50%
3 Months	70.00%	49.10%	50.90%
4 Months	54.50%		
5 Months	43.20%	60.30%	39.70%
6 Months	28.90%		

**Fig. 2:**
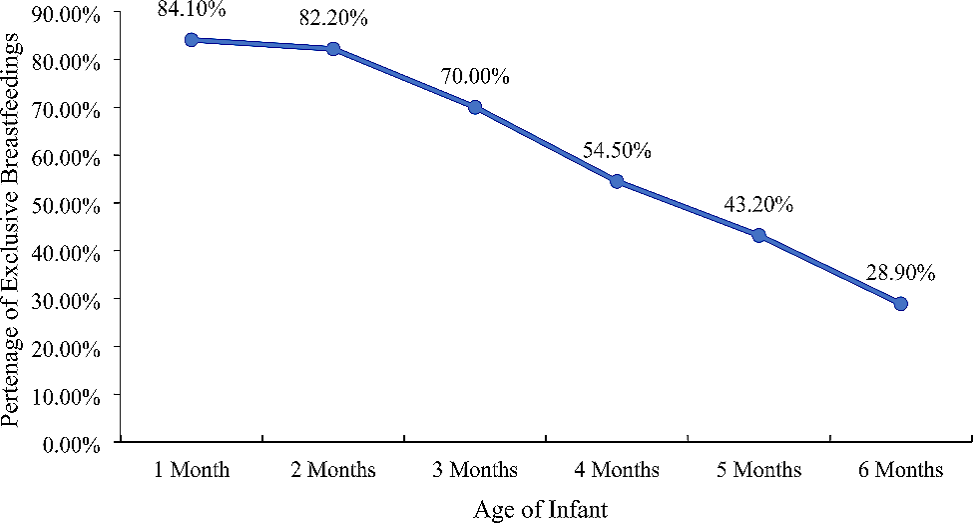
Percentages of Exclusive breastfeeding (EBF) by age of children

However, Fig. 2 shows the statistics that almost 84.1% of children during their first month of birth had the EBF, 54.5% amongst infants during the 3rd month of birth, and it has further degenerated to 28.9% amongst the infants while reaching their sixth month of birth.

**Fig. 3:**
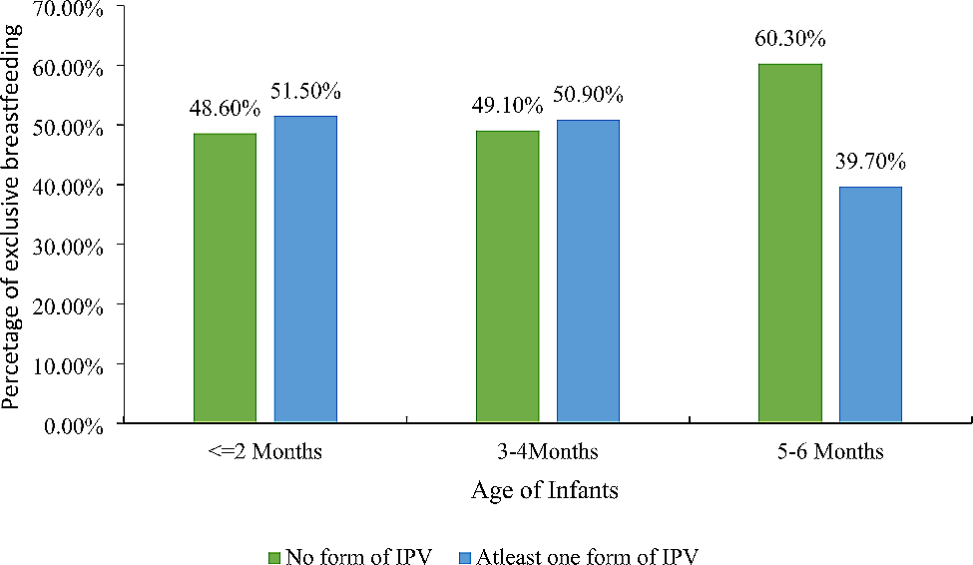
Percentage of Exclusive breastfeeding, maternal IPV, and age of child’s

Additionally, Fig. 3 depicted that the ratio of the EBF practice between females who have practiced any form of IPV does not vary for the infants who were 2 months or less and infants who were aged 3 to 4 months; the ratio of non-EBF practice has been greater amongst infants of the age of 5 to 6 months.

### Results of regression analysis

The regression investigation scrutinized the connotation among the types of IPV and EBF practices (EBF vs. mixed feeding), whereas modifying for the control factors. In the comprehensive case investigation, the respondents who have testified suffering from physical IPV have 33% (aOR; 0.68; 95% CI; 0.50, 0.99; P value < 0.05) decrease the odds of EBF practice while linked amongst those defendants who didn’t. Similarly, the respondent’s education and child age also depicted a significant relationship that decreases the chances of EBF with IPV (Table 5, Model 2). Correspondingly, an entity upsurge in the occurrence of IPV has related to the 5% (aOR; 0.85; 95% CI; 0.89, 0.99; P value < 0.05) decreased chances of EBF practices. Even though the practice of emotional, sexual, and collective items (IPV of any type) has revealed the propensity of condensed chances of EBF practices, nonetheless, the paraphernalia has been not arithmetically momentous at the 95% confidence interval for Models 1, 3, 4 in Table 5.

### Result of imputed regression analysis

The outcomes of the imputed regression have alike directions, whereas, dissimilar in extent. Furthermore, numerical connotation has been reserved for emotional IPV and the collective item (IPV of any type). The respondents conveyed suffering psychological IPV was 36% (AOR; 0.69, 95% CI; 0.50, 1.02; P value < 0.05) condensed possibility of EBF exercise while compared to respondents who didn’t practice EBF (Table 6 model 1). Despite the fact, the respondents who were informed undergoing any IPV have 29% (aOR; 0.78, 95% CI; 0.56, 1.09, P value < 0.05) abridged odds of EBF exercise while comparing alongside those respondents who have not exercised EBF (Table 6 model 4). Additionally, physical IPV outcome has been amplified commencing 34 to 39% (aOR; 0.73, 95% CI; 0.47, 0.89; P value < 0.05) (Table 6, Model 2), Table 6, model 5 have shown the outcome of a unit upsurge in the occurrence of IPV has been stayed unaffected.

**Table 5:** Weighted logistic regression relationship among intimate partner violence and exclusive breastfeeding (Comprehensive case)

**Variables**	**EBF, aOR, 95% Confidence Interval (CI)**				
	**M1**	**M2**	**M3**	**M4**	**M5**
Psychological IPV	0.63 (0.63,1.02)				
Physical IPV		0.68** (0.50,0.99)			
Sexual IPV			0.85 (0.59,1.50)		
Any IPV type				0.72 (0.59,1.09)	
IPV occurrence rate					0.85** (0.89,0.99)
Female’s Age	0.92 (0.92, 1.10)	0.94 (0.93, 0.98)	0.93 (0.94, 1.02)	0.96 (0.93, 1.01)	0.98 (0.93, 1.01)
Female’s Education	0.96** (0.94, 1.01)	0.98** (0.96, 1.01)	0.95** (0.95, 1.01)	0.98** (0.96, 1.01)	0.98** (0.93, 1.02)
Children age	0.62***(0.59, 0.67)	0.65*** (0.58, 0.68)	0.63***(0.58, 0.68)	0.68***(0.57, 0.69)	0.63*** (0.59, 0.69)
Employment (Ref = no)	1.08 (0.89, 1.29)	0.99 (0.99, 1.31)	1.07 (0.89, 1.36)	1.36 (0.72, 1.33)	1.07 (0.88, 1.35)
No of children’s (Ref = Primiparous)	0.85 (0.59, 1.18)	1.18 (0.90, 1.08)	0.82 (0.60, 1.17)	1.19 (1.00, 1.09)	0.84 (0.59, 1.16)
Education (Husband)	1.04 (0.98, 1.00)	0.99 (0.98, 1.00)	1.04 (0.98, 1.04)	1.04 (1.00, 1.09)	1.04 (0.99, 1.05)
Rural (Ref = urban)	1.21 (0.60, 1.60)	1.22 (0.74, 1.61)	1.22 (0.74, 1.61)	1.21 (0.79, 2.33)	0.84 (0.59, 1.20)
Family wealth Index	1.05 (0.84, 1.28)	1.04 (0.84, 1.25)	1.02 (0.85, 1.28)	1.00 (0.99, 1.00)	1.03 (0.89, 1.19)
Nos. of under-five children	1.04 (0.93, 1.19)	1.04 (0.93, 1.19)	1.04 (0.93, 1.19)	1.04 (0.93, 1.19)	1.04 (0.93, 1.19)
Constants	13.28*** (7.18, 28.44)	13.31*** (7.31, 28.39)	13.51*** (7.19, 28.55)	13.43*** (7.41, 29.50)	13.21*** (7.21, 28.84)
Observations	1391	1391	1391	1391	1391

Sampling referred weights; *** P value < 0.01, ** P value < 0.05, aOR; Adjusted odd ratios, Under-five children’s; number of child’s less than five years of age

**Table 6:** Weighted logistic regression relationship among intimate partner violence and exclusive breastfeeding (Multiple Imputation)

**Variables**	**EBF, aOR, 95% Confidence Interval (CI))**				
	**M1**	**M2**	**M3**	**M4**	**M5**
Psychological IPV	0.69** (0.50,1.02)				
Physical IPV		0.73** (0.47,0.89)			
Sexual IPV			0.92 (0.59,1.39)		
Any IPV Type				0.78** (0.56,1.09)	
IPV occurrence rate					0.92** (0.89,1.01)
Female’s Age	0.92 (0.92, 1.00)	0.98 (0.93, 0.98)	0.93 (0.94, 1.00)	0.96 (0.93, 1.00)	0.98 (0.93, 0.99)
Female’s Education	0.98** (0.94, 0.99)	0.98** (0.96, 0.99)	0.98** (0.95, 0.99)	0.98** (0.96, 0.99)	0.98** (0.92, 0.99)
Children age	0.62***(0.59, 0.67)	0.65*** (0.58, 0.68)	0.63***(0.58, 0.68)	0.68***(0.57, 0.69)	0.63*** (0.59, 0.69)
Employment (Ref = No)	1.08 (0.88, 1.39)	0.99 (0.98, 1.00)	1.07 (0.90, 1.38)	1.36 (0.72, 1.33)	1.07 (0.89, 1.39)
No of children’s (Ref = Primiparous)	0.85 (0.59, 1.19)	1.18 (0.90, 1.08)	0.82 (0.58, 1.06)	1.19 (1.00, 1.09)	0.84 (0.59, 1.16)
Spouse Education	1.04 (0.98, 1.04)	0.99 (0.99, 1.00)	1.04 (0.98, 1.06)	1.04 (1.00, 1.09)	1.04 (1.00, 1.04)
Rural (Ref = urban)	1.21 (0.60, 1.60)	1.22 (0.74, 1.61)	1.22 (0.74, 1.61)	1.21 (0.79, 2.33)	0.84 (0.59, 1.21)
Family wealth Index	1.05 (0.84, 1.28)	1.04 (0.84, 1.25)	1.02 (0.85, 1.28)	1.00 (0.99, 1.00)	1.03 (0.89 1.19)
Nos. of under-five child’s	1.04 (0.93, 1.19)	1.04 (0.93, 1.19)	1.04 (0.93, 1.19)	1.04 (0.93, 1.19)	1.04 (0.93, 1.19)
Constants	13.28*** (7.18, 28.44)	13.31*** (7.31, 28.39)	13.51*** (7.19, 28.55)	13.43*** (7.41, 29.50)	13.21*** (7.21, 28.84)
Observations	1191	1191	1191	1191	1191

### Breastfeeding Initiation/Sensitivity analysis

To monitor the robustness of the outcomes of the current study, the author has conducted a Sensitivity analysis. Logistic regression has been utilized to examine the connotation among IPV and its types (physical, sexual, and physiological) and breastfeeding initiation (dichotomous: 1 = initiated, 0 = did not initiate), depicted in Table 7. The logistics regression analysis in each column demonstrates by what method every variable on the left side has been connected with breastfeeding initiation in all the models. The analysis displays that there is a significant connotation among physical IPV and breastfeeding initiation (aOR 0.78, 95% CI: 0.61-1.00), presenting that those womenfolk have bear the violence from their partners have the inferior likelihood of initiating breastfeeding in Table 7 Model 2. While comparing with Table 5, the connotation among IPV and EBF (Comprehensive case) the values of IPV and EBF (aOR; 0.68, 95% CI: 0.50–0.99) for the Table 5 model 2. The results of regression analysis for both complete case and sensitivity analysis have depicted those outcomes have the same direction but differ in magnitude. Similarly, an increase in the occurrence of IPV has also been linked with minimum chances of breastfeeding initiation (aOR; 0.88, 95% CI; 0.98, 1.00; P value < 0.05) in Table 7, model 5. Correspondingly, if we compare the comprehensive case connotation among EBF and IPV, the values of IPV and EBF (aOR; 0.73, 95% CI; 0.47–0.89) for Table 6 model 2 have also shown similar directions but different magnitudes. Nevertheless, the occurrence of the emotional, sexual, and collective item (IPV any type) presented the propensity of minimum chances of breastfeeding initiation. However, the results have not been influential at the 95% CI in Table 7, models 1, 3, and 4. Eventually, we can say that the outcomes for the logistic regression for breastfeeding initiation have the same direction but differ in magnitude.

**Table 7:** Logistic regression relationship among IPV and Breastfeeding Initiation

**Variables**	**Breastfeeding Initiation, aOR, 95% confidence interval (CI)**				
	**M1**	**M2**	**M3**	**M4**	**M5**
Psychological IPV	0.92 (0.61,1.39)				
Physical IPV		0.78** (0.61,1.00)			
Sexual IPV			0.81 (0.42,1.33)		
Any IPV type				0.73 (0.48,1.09)	
IPV frequency score					0.88** (0.98,1.00)
Female’s Age	0.92 (0.92, 0.98)	0.98 (0.93, 0.98)	0.93 (0.94, 1.01)	0.96 (0.93, 1.00)	0.98 (0.93, 1.01)
Female’s Education	0.96** (0.94, 0.99)	0.98** (0.96, 0.99)	0.95** (0.95, 0.98)	0.98** (0.95, 0.99)	0.98** (0.93, 1.00)
Children age	0.62***(0.59, 0.67)	0.65*** (0.58, 0.68)	0.63***(0.58, 0.68)	0.68***(0.57, 0.69)	0.63*** (0.59, 0.69)
Employment (Ref = No)	1.08 (0.89, 1.29)	0.99 (0.99, 1.00)	1.07 (0.85 1.39)	1.36 (0.72, 1.33)	1.07 (0.88, 1.34)
Nos of children (Ref = Primiparous)	0.85 (0.62, 1.08)	1.18 (0.90, 1.08)	0.82 (0.60, 1.09)	1.19 (1.00, 1.09)	0.84 (0.59, 1.16)
Spouse Education	1.04 (0.98, 1.02)	0.99 (0.98, 1.00)	1.04 (0.98, 1.03)	1.04 (1.00, 1.09)	1.04 (0.98, 1.01)
Rural (Ref = urban)	1.21 (0.60, 1.60)	1.22 (0.74, 1.61)	1.22 (0.74, 1.61)	1.21 (0.79, 2.33)	0.84 (0.59, 1.21)
Family wealth Index	1.05 (0.84, 1.28)	1.04 (0.84, 1.25)	1.02 (0.85, 1.28)	1.00 (0.99, 1.00)	1.03 (0.89, 1.29)
Nos. of under-five children	1.04 (0.93, 1.19)	1.04 (0.93, 1.19)	1.04 (0.93, 1.19)	1.04 (0.93, 1.19)	1.04 (0.93, 1.19)
Constants	13.28*** (7.18, 28.44)	13.31*** (7.31, 28.39)	13.51*** (7.19, 28.55)	13.43*** (7.41, 29.50)	13.21*** (7.21, 28.84)
Observations	1191	1191	1191	1191	1191

Sampling referred weights

*** P value < 0.01

** P value < 0.05

aOR; Adjusted odd ratios, Under-five children’s; the number of children less than five years of age

## Discussion

Through the utilization of PDHS Dataset 2018, the current research has scrutinized the connotations amongst IPV and the EBF practices of nurturing mothers in Pakistan. According to the current research outcomes, the case-wise omitting of the observations in the comprehensive case investigation has weakened the outcome of the current connection. The imputed investigation recommends that maternal IPV practiced during the pregnancy or postpartum interval has been connected with suboptimal practices of EBF. Excluding sexual IPV, the other 2 types of motherly IPV (psychological and physical) have been adversely linked with EBF practices, alongside physical IPV presenting a greater extent. Additionally, the current research outcomes have also recommended that a measured practice of maternal IPV has a substantial connotation through suboptimal breastfeeding. It has been pointed out that numerous types or frequent occurrences of IPV around pregnancy or post-delivery time have been connected to suboptimal breastfeeding of early babies. The present research has also added to information by presenting how dissimilar types of IPV experienced through a period of pregnancy or post-delivery have been connected through EBF of new babies in Pakistani background. According to our information based upon the facts, the current association had not been formerly scrutinized, having an emphasis on IPV practiced during the gestation period or post-delivery.

The outcomes of the current research have been different from the outcomes of [31], who utilized the DHS dataset and were informed that maternal IPV had no connotation to the EBF in Pakistan. Nevertheless, the current dissimilarity has been possibly subjected to two vital elements. Initially, concerning their research, they utilized a different dataset, whereas, in the context of Pakistan, we have utilized a different and latest DHS Dataset. Furthermore, their research had intellectualized IPV as a lifetime practice, whereas, in the current research, the researcher has hypothesized that it has been practiced during the pregnancy or post-delivery period. It has been recommended that the vicinity of the violence associated with the breastfeeding period might have a vital influence in defining a connotation. Although actions that have taken place for an extended period might or might not be linked to the mother’s capability and wish to feed the infant, a violent action that has been practiced during the period of pregnancy or post-delivery is probable to the expected outcome. Likewise, both emotional and physical IPV were connected to suboptimal breastfeeding. The current outcome replicates the lacking supposition [43, 44], which has been similar through additional cross-sectional research in Bangladesh, the USA, and India [7, 32, 45]. Mothers who have experienced IPV might be little likely to breastfeed their children, ideally as an effect of physical or emotional inequality [46]. The track by which this might occur can be of various types. Primarily, females who are victims of IPV have been informed that they are at more threat of depressing signs that could take them to further particular risk actions such as drinking alcohol, smoking, or drug addiction. Substance exploitation has been connected to the initial stage of termination from breastfeeding practice either due to the probable threat to the infants or showing carelessness in caring responsibilities [47, 48].

Concerning sexual IPV, our results have pointed out that nurturing moms who have suffered sexual violence have been more likely to exercise EBF compared to those mothers who have been informed not to suffer from sexual violence. Nevertheless, the current findings of our research have been matched to the investigation of [45]. The studies of LMIC countries have shown dissimilar results through examinations where they utilized the collective statistics of population-based research diagonally [49]. A comparatively lesser number of explanations through the current cluster in existing research might influence the outcomes. Furthermore, it was recognized that physical violence in close connections is more probable to happen through emotional exploitation instead of sexual exploitation [50]. This might be a situation of difference broadcasting bias through the traditional ins and outs. Although the current investigation has been cautiously formed together with the existing practices, it has also been pretested through apprehending the information of rape and sexual violence till postures a moral and operational contest. Another issue is the quiet culture concerning the occurrence of rape through the subsequent stigmatization [51, 52]. One more main cause is the Pakistani culture, which has been categorized by male supremacy. It has been supposed to generate certain concepts of male sexual power, and as an outcome females might be less likely to ^,^ bear undesirable sex as an action of violence [19].

## Conclusions

The current research has been offering novel outcomes from the perspective of Pakistan, presenting that IPV practices, predominantly emotional and physical violence during pregnancy and postpartum time frame, have an adverse connotation with the probability of EBF for infants that are under 6 months. The strategy implications have been arising on inspiring a structure not marking out sufferers of sexual exploitation, in the contact that “culture of silence” has not forced them to agonize in quietness. Despite the fact that longitudinal investigation might still be required to benefit the superior understanding of this connection, forthcoming investigations must also attempt to disconnect abuse practiced around the time of pregnancy and postpartum so that upcoming examinations must focus on distinguishing the extent of connotation for both. Our findings suggested that government policy-making institutions should focus on programs that help in the reduction of IPV practices in Pakistan.

### Limitation of the study

Nevertheless, the current investigation has a few limitations linked to the investigation and study design. First of all, the utilization of cross-sectional statistics with similar research designs made it challenging for any kind of assertion of fundamental associations. Furthermore, the current study utilizes the examination of variables, which was limited to what was apprehended by the assessment. Precisely, variables regarding (post-delivery) cheerless indicators were not taken. If it has been taken, the intermediating role must have been scrutinized. Thirdly, throughout the examination, violent practices were taken as actions that occurred during the last 12 months. There has been no disconnection among the actions that occurred throughout the pregnancy or post-delivery. Due to this outcome, the investigation has been controlled from this contradiction. Fourthly, with respect to the fauna of the consequence item of concentration, bidirectional enactment of the violence has not been measured during the procedure of the operationalization of IPV. As offenders, it might not be probable to stop a female from breastfeeding even after they become the sufferer. Finally, EBF practice has been constructed on point-in-time calculation (24-h recollection). It may create the possibility of introducing certain biases in the statistics; in the meantime, the infants might have been nourished through non-recommended foodstuff in a prior time frame but have not been nourished during the last 24 h.

## Data Availability

The datasets used for analysis and reaching the conclusions of this study are available online at MEASURE DHS (https://www.dhsprogram.com/data/availabledatasets.cfm). It is released upon request, subject to approval.

## Abbreviations

AOR: Adjusted Odd Ratio
PDHS: Pakistan Demographic and Health Survey
EBF: Exclusive Breastfeeding practices
IPV: Intimate Partner Violence
MAR: Missing at random
PWA: Pakistan Women’s Association
